# Supplementary material for: Appropriate complementary feeding practice and associated factors among mothers of children aged 6–23 months in Bhimphedi rural municipality of Nepal
Source: PLoS One. 2024 Mar 6;19(3):e0299969. doi: 10.1371/journal.pone.0299969 (PMC10917259; doi:10.1371/journal.pone.0299969)
Supplement: Attachment — Submitted filename: Response to Reviewers.docx [file pone.0299969.s002.docx]

Naveed Sadiq

Academic Editor

PLOS ONE

Dear Naveed,

We thank you and the reviewers for a thorough reading and constructive feedback on our manuscript (PONE-D-23-31826) and for the opportunity to improve the original article. We are pleased to submit the revised research article titled, “Appropriate complementary feeding practice and associated factors among mothers of children aged 6-23 months in Bhimphedi rural municipality of Nepal” for your consideration in the PLOS ONE.

On behalf of my author and co-author, we are sending the revised manuscript with track change and the revised manuscript. We believed that we had addressed all the comments from reviewers and hope that the revised manuscript is acceptable for publication in PLOS ONE.

On the following pages, you will find our response to the reviewer's comments. We thank you for your consideration of this resubmission.

We appreciate your time and look forward to your response.

Sincerely,

Anil K.C. (corresponding author)

kcanil707@gmail.com

+977-9847907142

Journal requirements:

1. When submitting your revision, we need you to address these additional requirements.

Please ensure that your manuscript meets PLOS ONE's style requirements, including those for file naming. The PLOS ONE style templates can be found at 
<https://journals.plos.org/plosone/s/file?id=wjVg/PLOSOne_formatting_sample_main_body.pdf>  and <https://journals.plos.org/plosone/s/file?id=ba62/PLOSOne_formatting_sample_title_authors_affiliations.pdf>.

Response: Rechecked

2. Please include your full ethics statement in the ‘Methods’ section of your manuscript file. In your statement, please include the full name of the IRB or ethics committee who approved or waived your study, as well as whether or not you obtained informed written or verbal consent. If consent was waived for your study, please include this information in your statement as well.

Response: Full ethics statement is included in method section under Ethical Consideration section.

3. We are unable to open your Supporting Information file [Supporting Information.sav]. Please kindly revise as necessary and re-upload.

Response: Supporting Information file is SPSS data file. I have re-uploaded that file. I have also shared that file on figshare https://doi.org/10.6084/m9.figshare.24633153.v1.

4. Please review your reference list to ensure that it is complete and correct. If you have cited papers that have been retracted, please include the rationale for doing so in the manuscript text, or remove these references and replace them with relevant current references. Any changes to the reference list should be mentioned in the rebuttal letter that accompanies your revised manuscript. If you need to cite a retracted article, indicate the article’s retracted status in the References list and also include a citation and full reference for the retraction notice.
Response: Checked. We have not cited papers that have been retracted.

Review Comments to the Author
Reviewer #1: 1. Line (L) 61-62: WHO recommended to continued breastfeeding up to 2 years of beyond not 12 months as stated. Any comment or justification on author's statement on this matter?
Response: This is the estimated contribution of breastfeeding (Exclusive breastfeeding in the first 6 months of life and continued breastfeeding from 6 to 11 months) and complementary feeding on the prevention of under five deaths.
2. L61-65: The statement is not clear. Please paraphrase the statement to be clearer and what are on basis of this statement?

Response: Paraphrased

3. L88-89: I would suggest the author to consider to follow and standardised the indicator's name according to WHO Unicef IYCF indicator 2021. 'Introduction of solid, semi-solid or soft food 6-8 months (ISSSF)

Response: “Complementary feeding” indicator is replaced with “Introduction of solid, semi-solid or soft food 6-8 months (ISSSF)”

4. L113-119: I would recommend the author to elaborate more on the selection of 3 indicators from 17 IYCF WHO IYCF practice's indicators. To justify why choose the three selected indicators and how classified it as Appropriate Complementary Feeding Practices. The term of 'core indicator' was used in WHO IYCF Practices Indicators 2008 and not in WHO IYCF Practices Indicators 2021 as the revised version divided into 3 main indicators (Breastfeeding, Complementary Feeding and Other practices indicator) rather than 2 main indicator in 2008 (Core vs Optional indicators)

As ISSSF become one of the three indicator to determine prevalence of ACFP, how the author analyse data as the ISSSF data is meant for the children aged 6-8 months (age group to assess the indicator). Can the author explain on how author assess this indicator? If the denominator was the children age 6-23 months, author need to reanalyse the data.
Response: Necessary changes were made.

The research participants of this study were the mothers of children of age 6-23 months. The information regarding ISSSF was collected retrospectively from all the respondent’s whether they initiated introduction of solid, semi-solid or soft food at the age of 6-8 month. The denominator in this indicator contains the information of ISSSF when the age of the children was 6-8 months.
5. L141-143: Rephrase in the better way to explain the definition of the topic.
Response: Previous operation definition is changed to “The proportion of children age 6–23 months who had been introduced to solid, semi-solid, or soft foods at the age between 6 to 8 months.”
6. L239-240: The indicator was not follow the definition of WHO IYCF Practices Indicator 2021 (ISSSF). If the author do not want to follow the indicator's definition, the author need to explain and elaborate more on this topic.

Response: Operation definition of ISSSF is revised to “The proportion of children age 6–23 months who had been introduced to solid, semi-solid, or soft foods at the age between 6 to 8 months”
Figure 1: Fig 1 showed the ISSSF indicator data come from the total respondent which is not follow the WHO guideline to asses the IYCF indicators. I assumed the author used the definition as states at line 141-143 and using the WHO IYCF Indicators for assessing infant and young child feeding practices. Standardise in term of definition is important especially to compare the result among the research on this topic.

Response: Operation definition was changed. Even though, almost all study cited the standard definition of WHO guideline, they have calculated ISSSF by taking total respondents, so we can compare the result with these studies.

Reviewer #2: 1. As per the measure in methodology section, appropriate complementary feeding practice is defined as indicator three of the WHO core IYCF i.e. timely introduction of solid, complementary feeding minimum dietary diversity and minimum meal frequency. But in the result section, appropriate complementary feeding practice and all these indicators presented separately. So be clear about the measurement. If you need any correction, correct both in the abstract result section and main findings.

Response: All three indicators along with Appropriate complementary feeding practice was presented in the figure because those indicator has equal importance in Infant and Young Child Nutrition and will also give readers comprehensive picture from which ACFP is derived.
2. Better to specify the "to address associated factors" in conclusion section of abstract.

Response: Associated factors are presented in result section.

3. What is basis for classification of monthly income? Better use any standard basis

Response: Monthly income was not classified in any standard basis because authentic data related to average monthly income of general population is lacking.

4. Found missing figure 1 in results section

Response: Figure was uploaded separately

5. it would be more clear for reader if you include fig. or table related to descriptive findings i.e. timing of introduction of solid, complementary feeding minimum dietary diversity and minimum meal frequency in results section.

Response: As per the guideline of PLOS ONE, all figures should be uploaded separately. So, figure was uploaded separately.
