# Supplementary material for: Appropriate complementary feeding practice and associated factors among mothers of children aged 6–23 months in Bhimphedi rural municipality of Nepal
Source: PLoS One. 2024 Mar 6;19(3):e0299969. doi: 10.1371/journal.pone.0299969 (PMC10917259; doi:10.1371/journal.pone.0299969)
Supplement: Attachment — Submitted filename: Response to Reviewers.docx [file pone.0299969.s003.docx]

Naveed Sadiq

Academic Editor

PLOS ONE

Dear Naveed,

We thank you and the reviewers for a thorough reading and constructive feedback on our manuscript (PONE-D-23-31826) and for the opportunity to improve the original article. We are pleased to submit the revised research article titled, “Appropriate complementary feeding practice and associated factors among mothers of children aged 6-23 months in Bhimphedi rural municipality of Nepal” for your consideration in the PLOS ONE.

On behalf of my author and co-author, we are sending the revised manuscript with track change and the revised manuscript. We believed that we had addressed all the comments from reviewers and hope that the revised manuscript is acceptable for publication in PLOS ONE.

On the following pages, you will find our response to the reviewer's comments. We thank you for your consideration of this resubmission.

We appreciate your time and look forward to your response.

Sincerely,

Anil K.C. (corresponding author)

kcanil707@gmail.com

+977-9847907142

Journal requirements:

Please review your reference list to ensure that it is complete and correct. If you have cited papers that have been retracted, please include the rationale for doing so in the manuscript text, or remove these references and replace them with relevant current references. Any changes to the reference list should be mentioned in the rebuttal letter that accompanies your revised manuscript. If you need to cite a retracted article, indicate the article’s retracted status in the References list and also include a citation and full reference for the retraction notice.

Response: Checked. We have not cited papers that have been retracted.

**Review Comments to the Author**

Reviewer #1: May i suggest to the author to remove Lines 229-230 as it is repetitive?

And Good luck to the author

Response: Removed

Thank you

Reviewer #2: Authors probably misunderstood the previous round comment 1. I think the study's outcome variable is appropriate complementary feeding practice (ACFP). They have been measured ACFP by using a composite indicator comprising three of the WHO core IYCF indicators i.e. timely introduction of solid, complementary feeding, minimum dietary diversity, and minimum meal frequency. But in abstract results are not so clear therefore, kindly please go through the main finding's result section and write in that way. I accept that the three indicators are equal important but the comment is just for writing style and clarity.

Response: Necessary changes have been made in abstract
